# Optimal Tunnel Positioning and Graft Diameter to Minimize Impingement in Single-Bundle ACL Reconstruction: A 3D CT Simulation Analysis

**DOI:** 10.3390/medicina61060946

**Published:** 2025-05-22

**Authors:** Sang-Woo Jeon, Sung-Hwan Kim, Kang-Il Kim

**Affiliations:** 1 Department of Orthopedic Surgery, Ewha Womans University Seoul Hospital, Seoul 07804, Republic of Korea; 2 Department of Orthopedic Surgery, Severance Hospital, College of Medicine, Yonsei University, Seoul 03722, Republic of Korea; 3 Department of Orthopedic Surgery, Kyung Hee University Hospital at Gangdong, 149 Sangil-dong, Gangdong-gu, Seoul 05278, Republic of Korea

**Keywords:** anterior cruciate ligament reconstruction, tunnel positioning, graft impingement, three-dimensional simulation, knee biomechanics

## Abstract

Background and Objectives: Graft impingement against the intercondylar notch has been identified as a significant contributor to graft deterioration and suboptimal outcomes following anterior cruciate ligament (ACL) reconstruction. This study aimed to (1) identify the optimal combination of tunnel positions that minimizes impingement between the ACL graft and femoral intercondylar notch. Materials and Methods: Three-dimensional models of nine normal knees were reconstructed using computed tomography scans obtained at four knee flexion angles (0°, 45°, 90°, and 120°). Virtual ACL grafts with diameters of 7 mm and 9 mm were modeled as cylinders. Nine graft configurations were investigated by varying femoral and tibial footprint locations (anteromedial, central, and posterolateral) in all possible combinations. For each configuration, impingement volume was quantified by measuring the overlap between the intercondylar notch and the virtual graft using Boolean operators in 3D simulation software. The effects of graft diameter, footprint location, and knee flexion angle on impingement volume were analyzed. Results: Maximum impingement volumes were observed at 0° knee extension, with significant reductions at 45° flexion (*p* < 0.01) and negligible impingement at 90° and 120° flexion. The 9 mm diameter grafts demonstrated significantly greater impingement volumes than 7 mm grafts (*p* < 0.01). Impingement volumes increased progressively as footprint locations shifted from posterolateral to anteromedial positions in both femoral and tibial components. However, statistically significant differences in impingement volume across footprint locations were observed only for tibial positioning (*p* < 0.001), not for femoral positioning (*p* > 0.05). The femoral anteromedial-tibial anteromedial configuration exhibited the highest impingement volume (577.8 ± 171.3 mm^3^ for 9 mm grafts), while the femoral posterolateral-tibial posterolateral configuration showed the lowest (73.5 ± 85.6 mm^3^). Conclusions: Tunnel position, graft diameter, and knee flexion angle significantly influence impingement risk in ACL reconstruction. Tibial tunnel position appears more critical than femoral position in minimizing graft impingement. Posterolateral positioning of tunnels, particularly on the tibial side, may reduce impingement volume. Clinical Relevance: This study provides quantitative evidence to guide surgeons in optimizing tunnel placement and graft selection for anatomical single-bundle ACL reconstruction, potentially reducing the risk of graft deterioration and failure due to mechanical impingement.

## 1. Introduction

As the incidence of anterior cruciate ligament (ACL) reconstructions continues to rise alongside sustained demands for high-level athletic performance, the frequency of revision ACL reconstruction is expected to increase accordingly [1,2]. Anterior cruciate ligament (ACL) reconstructions have been reported to fail in approximately 12% of cases [3]. Among the technical causes of failure, femoral tunnel malposition is the most frequently cited, accounting for 63% to 90% of such cases [4]. Additionally, graft impingement against the intercondylar notch has been identified as a significant contributor to graft deterioration and postoperative limitations in range of motion [5].

Graft impingement can lead to several complications, including anterior knee pain, joint effusions, loss of extension, and recurrent instability. Anterior knee pain typically results from the graft impinging against the intercondylar notch when the knee approaches full extension. Effusions may develop due to wear particles and fractured graft fibers caused by graft abrasion. Loss of extension can occur when the graft acts as a checkrein against the intercondylar notch. In cases of severe impingement, graft failure may ensue, ultimately leading to recurrent instability [5,6].

There is some variability among previous studies regarding the precise localization of the anatomic center of the ACL femoral footprint [7,8]. The anatomy of the ACL footprint exhibits considerable variability in both size and shape [9]. Therefore, the optimal tunnel position in anatomic single-bundle ACL reconstruction may vary depending on the graft diameter as well as the size and morphology of the ACL footprint [10,11]. With the development of surgical techniques that allow independent creation of the femoral tunnel from the tibial tunnel, it has become possible to minimize graft impingement by considering the morphology of the femoral notch, as well as the femoral and tibial footprints [11]. Placement of the femoral tunnel in a more proximal and anterior position is correlated with a higher likelihood of graft impingement [12,13]. With regard to the tibial tunnel, graft impingement in full knee extension remains a concern associated with anterior tunnel placement. However, a limited number of biomechanical and clinical studies have demonstrated that anterior tibial tunnel placement can improve stability without compromising knee extension [14,15,16].

The purpose of the present study was to identify the optimal combination of tibial and femoral tunnel positions that minimizes impingement between the ACL graft and the femoral intercondylar notch in anatomical single-bundle ACL reconstruction. It was hypothesized that more anterior positioning of both the tibial and femoral tunnels would be associated with increased impingement volume.

## 2. Materials and Methods

### 2.1. 3D Model Reconstruction

Computed tomography (CT) images of the knee from nine subjects were included in this study. The nine knee models consisted of six males and three females. Three-dimensional CT scans were acquired using a 16-channel CT scanner (GE Healthcare, Chicago, IL, USA) with 0.625 mm slice thickness at four distinct knee flexion angles (0°, 45°, 90°, and 120°) in healthy volunteers. Subjects were selected based on the following inclusion criteria: (1) absence of femoral or tibial fractures; (2) no osseous deformities; (3) no ligamentous injuries; (4) no history of surgical intervention; and (5) absence of osteoarthritis exceeding Kellgren-Lawrence grade I [17]. Digital Imaging and Communications in Medicine (DICOM) data were extracted from the picture archiving and communication system (Centricity PACS, GE Medical System Information Technologies, Milwaukee, WI, USA). These data were subsequently imported into Mimics software (version 21.0; Materialise, Leuven, Belgium) for three-dimensional (3D) reconstruction of the femoral and tibial models.

**Figure 1:**
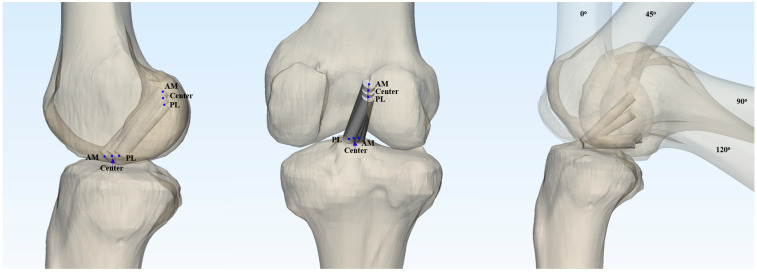
A total of 72 ACL cylinders were generated by combining three distinct femoral and tibial footprint locations, two graft diameters, and four knee flexion angles (0°, 45°, 90°, and 120°).

In the present study, the anterior cruciate ligament (ACL) graft was simplified to a virtual cylinder in the three-dimensional (3D) model. Based on previous research indicating that double-looped semitendinosus and gracilis grafts have an average diameter of 8 mm (range 7–9 mm) [18], virtual grafts with diameters of 7 mm and 9 mm were modeled. Nine different graft configurations were investigated by varying the femoral and tibial footprint locations (anteromedial, central, and posteromedial) in all possible combinations. Taking into account the two graft diameters and four knee flexion angles (0°, 45°, 90°, and 120°), a total of 72 virtual ACL cylinders were created and analyzed (Figure 1).

**Figure 2:**
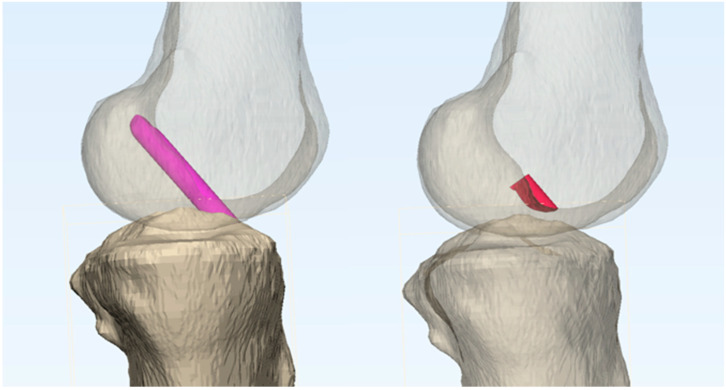
Impingement volume was represented as the overlap volume (shown in red in the right model) between the intercondylar notch of the femoral condyle and the virtual graft (depicted in magenta in the left model).

For each configuration, the impingement volume was quantified by measuring the overlap between the intercondylar notch of the femoral condyle and the virtual graft. This measurement was performed using Boolean operators in the Mimics 3D simulation program, which has been validated to provide measurement accuracy within 1 mm [19] (Figure 2).

**Figure 3:**
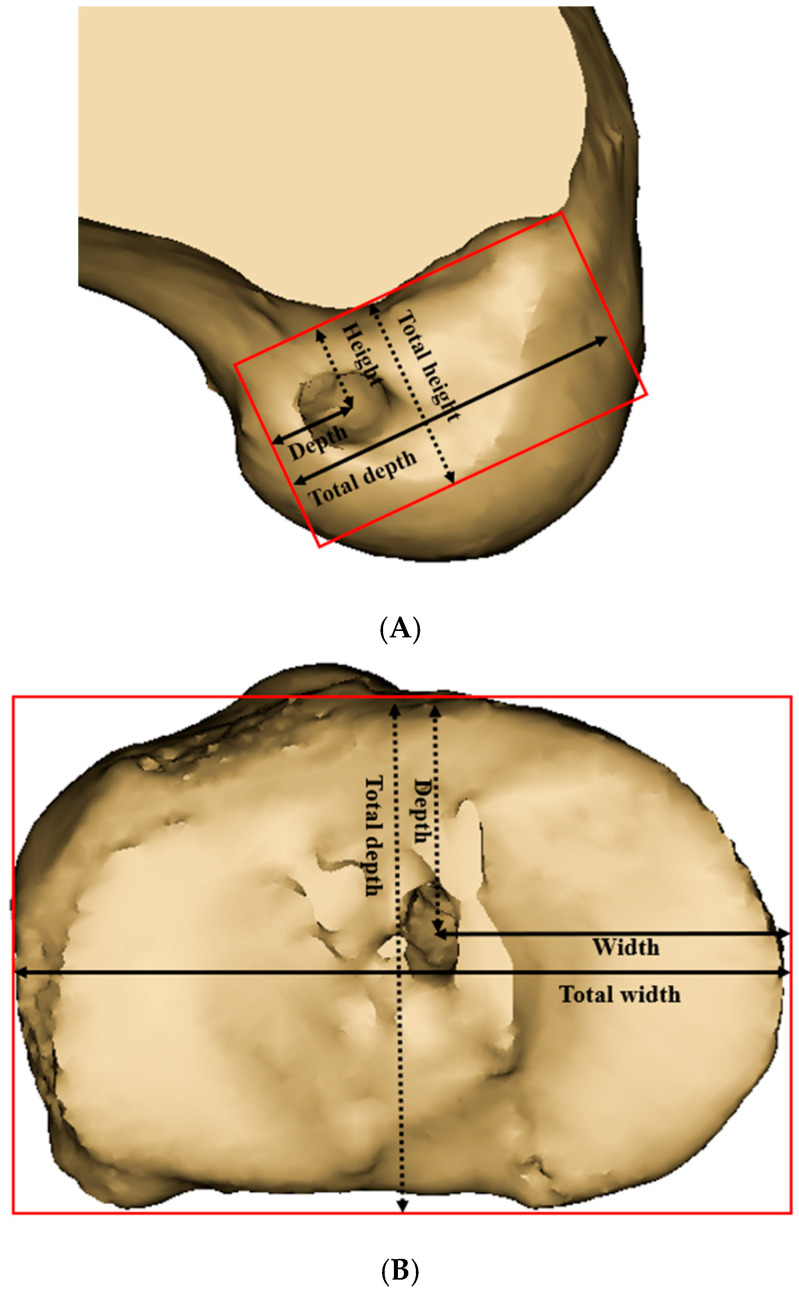
(**A**) The method for determining the femoral footprint location involved calculating the height as the ratio of the distance from the superior border of the reference frame to the footprint center (short dashed line) to the total height of the reference frame (long dashed line), multiplied by 100. Similarly, the depth was calculated as the ratio of the distance from the posterior border of the reference frame to the footprint center (short solid line) to the total depth of the reference frame (long solid line), multiplied by 100. (**B**) The method for determining the tibial footprint location involved calculating the depth as the ratio of the distance from the anterior border of the reference frame to the footprint center (short dashed line) to the total depth of the reference frame (long dashed line), multiplied by 100. Similarly, the width was calculated as the ratio of the distance from the medial border of the reference frame to the footprint center (short solid line) to the total width of the reference frame (long solid line), multiplied by 100.

The three-dimensional reconstructed femoral model was positioned in a true lateral view, with both femoral condyles superimposed. The medial femoral condyle was virtually removed from the 3D model to allow for a direct visualization of the medial wall of the lateral femoral condyle. The neutral position of the tibia, which provided an unobstructed view of the tibial joint surface, was established [20]. To define the centers of the ACL femoral and tibial footprints for the anteromedial (AM), central, and posterolateral (PL) bundles, we adopted the classical grid system proposed by Bernard et al. [21]. The reference frame was defined using a rectangular coordinate system, with the line passing through the highest point of the intercondylar notch as the superior border, and the outer margins of the lateral wall of the intercondylar notch serving as the lateral and medial borders (Figure 3A).

### 2.2. Footprint Positioning

According to previous studies using 3D CT models, the femoral center of the AM bundle footprint was identified at a point located 25% of the total height, measured from the superior to inferior border of the medial wall of the lateral femoral condyle, and 33% of the total depth, measured from the anterior to posterior border [22,23]. The femoral center of the PL bundle footprint was identified at a point located 45.1% of the total height and 50% of the total depth, measured in the same manner.

The reference frame was established using the joint surface in the tibial neutral position with a top view, and the cortical outline was defined as the outer border (Figure 3B). The tibial center of the AM bundle was located at a point 35% of the total depth (measured from the anterior-to-posterior border) and 50.5% of the total width (measured from the medial-to-lateral border). The tibial center of the PM bundle was located at a point 46.4% of the total depth and 52.4% of the total width [22,23]. The central point was defined as the midpoint of the line connecting the AM and PL bundle footprints. In both the tibia and femur, the central point was established at the midpoint between the AM and PL bundle footprints, as defined above.

### 2.3. Statistical Analysis

As this study was an experimental design, sample size calculation was not performed as nine pre-determined knee models were analyzed. Statistical analyses were conducted using SPSS Statistics version 23.0 (IBM Corp., Armonk, NY, USA). The mean impingement volume based on cylinder diameter was compared using an independent *t*-test. The mean impingement volume according to knee flexion angle was compared and analyzed using a paired *t*-test. The main effect of tibial and femoral footprint positions on impingement volume, based on data from 72 simulations across the nine knee models, was analyzed using one-way analysis of variance (ANOVA), comparing three types of footprints (AM, central, PL) for both tibia and femur [24]. The interaction effect between tibial and femoral footprint positions was analyzed using two-way analysis of variance (ANOVA) [25]. The level of significance was set at *p* < 0.05. Partial eta squared was calculated using post hoc tests.

## 3. Results

Table 1 presents the impingement volumes (mean ± standard deviation) for different graft configurations across various femoral and tibial footprint locations. Analysis was conducted for both 7 mm and 9 mm graft diameters. The width of the intercondylar notch was measured at its widest part from the anterior view of 3D reconstruction models of knees at 90 degrees of flexion, and the average value from nine models was 19.0 (±3.4) mm.

For the 7 mm graft diameter, the highest impingement volume was observed in the femoral anteromedial (AM)—tibial anteromedial (AM) configuration (338.3 ± 117.5 mm^3^), while the lowest was seen in the femoral posterolateral (PL)—tibial posterolateral (PL) configuration (26.6 ± 43.4 mm^3^). There was a statistically significant difference in impingement volumes across tibial footprint locations (*p* < 0.001), with AM positions consistently showing higher impingement than central and PL positions. However, differences between femoral footprint locations were not statistically significant (*p* = 0.174).

For the 9 mm graft diameter, impingement volumes were consistently higher compared to the 7 mm grafts. The femoral AM—tibial AM configuration again demonstrated the highest impingement volume (577.8 ± 171.3 mm^3^), while the femoral PL—tibial PL configuration showed the lowest (73.5 ± 85.6 mm^3^). Similar to the 7 mm graft results, significant differences were observed across tibial footprint locations (*p* < 0.001), but not across femoral footprint locations (*p* = 0.140).

Across all configurations, tibial footprint position had a greater influence on impingement volume than femoral footprint position. Moving from anteromedial to posterolateral positions on the tibia consistently resulted in significant reductions in impingement volume (*p* < 0.001).

**Figure 4:**
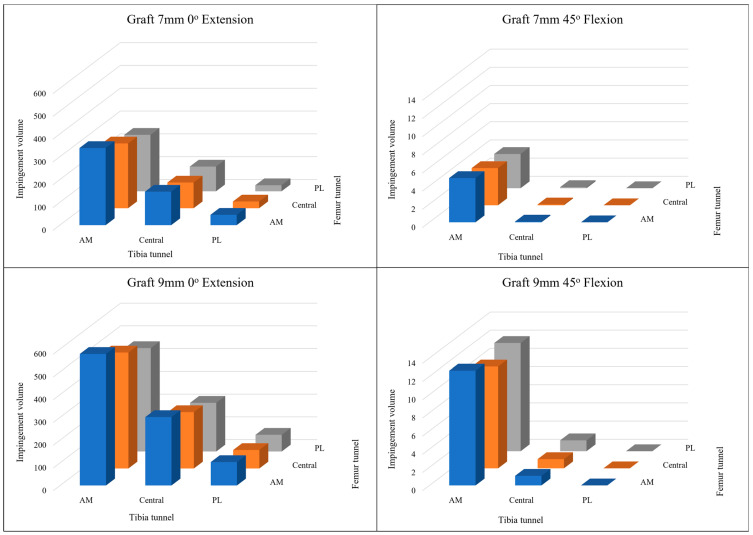
Impingement volume overlapping with the femur intercondylar notch of the cylinders according to two kinds of diameters and three kinds of footprint positions at 0-degree knee extension and 45-degree knee flexion.

Figure 4 illustrates the impingement volumes where the virtual ACL grafts overlap with the femoral intercondylar notch, comparing two different graft diameters (7 mm and 9 mm) across three distinct footprint positions at both 0° knee extension and 45° knee flexion.

At 0° knee extension, impingement volumes were consistently higher for the 9 mm diameter grafts compared to the 7 mm grafts across all footprint positions. The anteromedial footprint position demonstrated the greatest impingement volume, followed by the central position, with the posterolateral position showing the least impingement for both graft diameters.

When the knee was flexed to 45°, a notable reduction in impingement volume was observed across all configurations compared to full extension (*p* < 0.01). However, the pattern of impingement volume distribution remained consistent, with the anteromedial position continuing to exhibit the highest impingement values and the posterolateral position showing the lowest values.

At 90 degrees and 120 degrees of knee flexion, impingement volumes were negligible across all graft configurations.

This visualization in Figure 4 confirms that both graft diameter and footprint positioning significantly influence the degree of impingement, with larger diameter grafts and more anteriorly positioned footprints resulting in greater impingement volumes within the intercondylar notch.

## 4. Discussion

Graft impingement remains a significant challenge in anterior cruciate ligament (ACL) reconstruction, potentially leading to graft failure, limited range of motion, and poor clinical outcomes. Impingement occurs when the ACL graft contacts the intercondylar notch or roof during knee movement, causing mechanical stress and abrasion on the graft. While previous studies have examined various aspects of impingement, the combined effects of footprint location and graft diameter on impingement volumes have not been thoroughly investigated in three-dimensional models.

The main finding of this study is that impingement volume increases in a clear pattern as footprint locations shift from posterolateral to anteromedial positions, with this effect being particularly pronounced on the tibial side. Interestingly, while this trend was observed in both femoral and tibial footprints, statistical significance was only reached for variations in tibial positioning (*p* < 0.001). This suggests that tibial tunnel placement may be more critical than femoral placement in minimizing graft impingement, a finding that has important clinical implications for surgical technique.

The impingement volumes were substantially higher at full knee extension (0°) and decreased significantly at 45° of flexion, becoming negligible at 90° and 120° of flexion. This pattern reflects the biomechanical reality that ACL grafts are most vulnerable to impingement when the knee approaches full extension, which is consistent with previous literature [26,27]. The dramatic reduction in impingement at greater flexion angles indicates that postoperative rehabilitation protocols emphasizing early flexion exercises may help to minimize graft stress during the critical healing period.

Graft diameter also played a significant role in impingement volume, with 9 mm grafts demonstrating consistently higher impingement volumes compared to 7 mm grafts across all footprint combinations. A. Orsi et al. reported that larger ACL grafts as well as high femoral and anterior tibia insertion site shifting would lead to increased contact area and impingement forces [28]. The change in the amount of impingement according to the change of tibia was larger than that of the femur, which was statistically proven. It was confirmed that the location of the tibia is a key factor relative to the femur in terms of the impingement volume. This finding suggests that surgeons should consider graft size when planning tunnel positions, potentially adopting more posterior tunnel placements for larger diameter grafts to reduce impingement risk. However, this must be balanced against the mechanical advantage of larger grafts, which may offer superior strength and stability [29].

The femoral AM—tibial AM configuration demonstrated the highest impingement volume (577.8 ± 171.3 mm^3^ for 9 mm grafts), while the femoral PL—tibial PL configuration showed the lowest (73.5 ± 85.6 mm^3^). This nearly eight-fold difference highlights the critical importance of tunnel positioning in controlling graft impingement. These findings align with previous studies indicating that anterior placement of both femoral and tibial tunnels increases the risk of graft impingement [28,30,31,32].

It is noteworthy that while femoral footprint location variations did not reach statistical significance in affecting impingement volume, there was still a consistent trend of reduced impingement with more posterolateral positioning. This suggests that even small adjustments in femoral tunnel placement may contribute to reducing impingement risk, though the magnitude of this effect appears less substantial than that of tibial positioning. The anatomic center of the ACL femoral footprint exhibits considerable variability, with previous studies reporting variations of up to 6 mm proximally to distally and 2 mm anteriorly to posteriorly in ACL femoral origin size [7,9,33]. This natural diversity in footprint anatomy suggests that the optimal tunnel position may vary depending on individual patient anatomy, graft diameter, and original ACL footprint shape and size, particularly when employing anatomic single-bundle ACL reconstruction with independent tunnel drilling techniques. The findings from our study regarding minimization of graft impingement should be carefully considered when selecting tunnel positions to optimize graft longevity.

The clinical implications of our findings are substantial. Surgeons performing anatomical single-bundle ACL reconstruction should consider adopting more posterolateral tunnel positions, particularly on the tibial side, to minimize impingement risk. Additionally, in cases where larger diameter grafts are required, extra attention should be paid to tunnel positioning to avoid excessive impingement. Notchplasty may be considered for cases where anatomical positioning cannot avoid significant impingement, particularly with larger grafts [34].

Our study has several strengths, including the use of high-resolution 3D CT models from multiple subjects, evaluation across various knee flexion angles, and assessment of multiple footprint combinations with different graft diameters. The virtual simulation approach allowed for standardized measurements without the variability inherent in cadaveric studies or the ethical considerations of invasive testing in living subjects.

However, this study also has limitations. First, the simplified cylindrical model of the ACL graft does not fully capture the complex morphology and deformation characteristics of actual grafts. Second, our analysis focused solely on osseous impingement and did not consider soft tissue interactions or dynamic changes during functional activities. Third, while impingement volume provides a quantitative measure of potential graft-notch conflict, it does not directly translate to clinical outcomes or graft failure rates.

## 5. Conclusions

The impingement volume progressively increased as tunnel position shifted from posterolateral to anteromedial orientation in both tibial and femoral components in three-dimensional virtual simulation of anatomical ACL reconstruction. Maximum impingement occurred at full extension and with larger diameter grafts, while becoming negligible at flexion angles exceeding 90°. Strategic optimization of tunnel placement, with particular attention to tibial positioning, may significantly reduce impingement. Future research should explore the correlation between impingement volumes measured in virtual simulations and clinical outcomes such as graft failure rates, range of motion limitations, and patient-reported outcomes. Additionally, studies comparing the impingement characteristics of different graft types (e.g., bone-patellar tendon-bone versus hamstring) and fixation methods would provide valuable information for surgical planning.

## Figures and Tables

**Table 1:** Comparison of impingement volume at 0° Extension in Mimics simulation.

Graft Diameter 7 mm		Tibia (*p* < 0.001 ^b^)			*p*-Value
		AM	Central	PL	
Femur (*p* = 0.174 ^b^)	AM	338.3 ± 117.5	146.8 ± 111.5	45.4 ± 56.2	<0.001 ^a^
	Central	284 ± 125.1	112.6 ± 105.5	29.4 ± 45.2	<0.001 ^a^
	PL	247.6 ± 132.1	108.4 ± 96.7	26.6 ± 43.4	<0.001 ^a^
*p*-value		n.s. ^a^	n.s. ^a^	n.s. ^a^	
**Graft Diameter 9 mm**		**Tibia (*p <* 0.001 ^b^)**			***p*-Value**
		**AM**	**Central**	**PL**	
Femur (*p* = 0.140 ^b^)	AM	577.8 ± 171.3	300.8 ± 160.6	102.8 ± 105.2	<0.001 ^a^
	Central	509.2 ± 179.4	247.7 ± 156.2	81.2 ± 93.4	<0.001 ^a^
	PL	454.1 ± 183.5	214.1 ± 149.1	73.5 ± 85.6	<0.001 ^a^
*p*-value		n.s. ^a^	n.s. ^a^	n.s. ^a^	

AM Anteromedial, PL Posterolateral. ^a^ The value was obtained by the use of One-way analysis of variance. ^b^ The value was obtained by the use of Two-way analysis of variance.

## Data Availability

The original contributions presented in this study are included in the article. Further inquiries can be directed to the corresponding author.
